# Circular RNA in renal diseases

**DOI:** 10.1111/jcmm.15295

**Published:** 2020-04-25

**Authors:** Juan Jin, Haolu Sun, Chao Shi, Hui Yang, Yiwan Wu, Wanhai Li, Yu‐hang Dong, Liang Cai, Xiao‐ming Meng

**Affiliations:** ^1^ Department of Pharmacology Anhui Medical University Hefei China; ^2^ Department of Cardiac Surgery First Affiliated Hospital of Bengbu Medical College Bengbu City China; ^3^ The Key Laboratory of Major Autoimmune Diseases Anhui Institute of Innovative Drugs School of Pharmacy Anhui Medical University Hefei China

**Keywords:** biomarker, circRNA, exosome, fibrosis, renal disease

## Abstract

Circular RNA (circRNA) is a newly described type of non‐coding RNA. Active research is greatly enriching the current understanding of the expression and role of circRNA, and a large amount of evidence has implicated circRNA in the pathogenesis of certain renal diseases, such as renal cell carcinoma, acute kidney injury, diabetic nephropathy and lupus nephritis. Studies have found evidence that circRNAs regulate programmed cell death, invasion, and metastasis and serve as biomarkers in renal diseases. Recently, circRNAs were identified in exosomes secreted by the kidneys. Nevertheless, the function of circRNA in renal diseases remains ambiguous. Given that circRNAs are regulators of gene expression, they may be involved in the pathology of multiple renal diseases. Additionally, emerging evidence is showing that circulating circRNAs may serve as novel biomarkers for renal disease. In this review, we have summarized the identification, biogenesis, degradation, and functions of circRNA and have evaluated the roles of circRNA in renal diseases.

## 1 INTRODUCTION

Circular RNA (circRNA) is a type of long non‐coding RNA with covalently closed loops that are naturally resistant to exoribonuclease; therefore, it is predominantly located in the cytoplasm.^1^ CircRNAs are derived from non‐canonical splicing events and modulate gene expression and transcription via competition with endogenous RNAs. CircRNAs could be generated from introns, exons, untranslated regions or intergenic areas of the genome.^2^ According to the sequence, circRNA can be divided into three groups: exonic circular RNA (ecircRNA), which exclusively contains exons^1^; ciRNA, which is derived from intron lariats ^3^; and EIciRNA, which is composed of both exon and intron sequences.^4^ Although accumulating evidence has indicated that circRNAs act as miRNA sponges, they have been reported to act as sponges for RNA binding proteins (RBPs) and to modulate gene transcription^4^, ^5^; however, the functions of most circRNAs, especially in the renal system, remain poorly characterized.

Due to flawed methodologies, circRNAs have typically been regarded as by‐products of transcription with no function. However, in recent years, circRNAs have been identified as important regulators of multiple diseases, such as cancers and cardiovascular diseases.^6^, ^7^ Recently, increasing focus has been concentrated on the impact of circRNAs on the initiation and progression of renal diseases such as renal cancer, acute renal impairment, chronic nephritis and diabetic glomerular injury.^8^, ^9^, ^10^, ^11^ An increasing number of studies are finding the link between circRNAs and renal diseases, but results have been inconclusive. In this review, we introduce the relationship between circRNA and renal diseases. Research not only shows that circRNAs play critical roles in the progression of renal diseases but also that they may be used as new diagnostic biomarkers and therapeutic targets.

## 2 CHARACTERISTICS OF CIRCRNAs

### 2.1 Biogenesis of circRNAs

**FIGURE 1:**
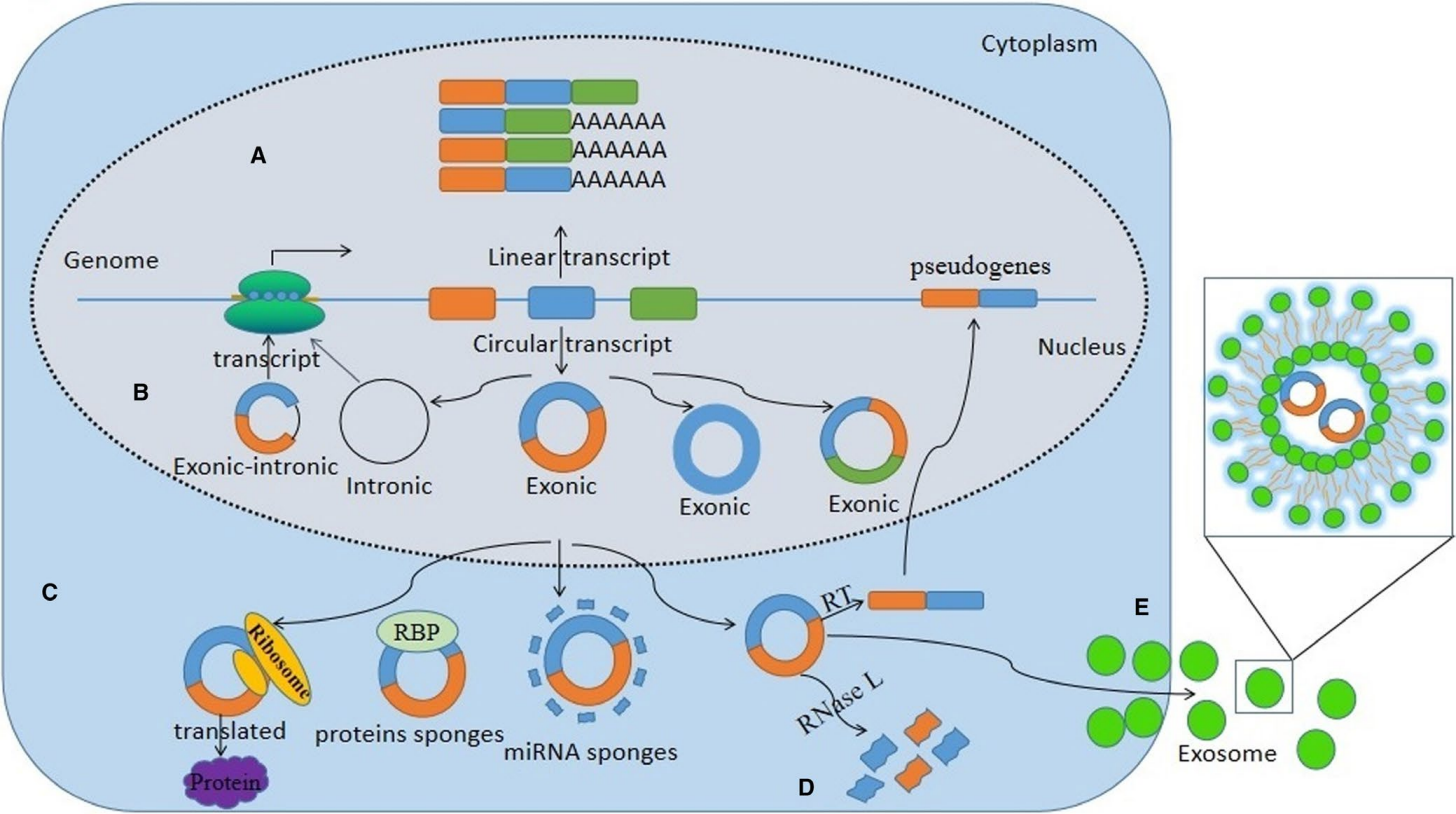
The biogenesis and functions of circRNAs. A, Pre‐mRNA is canonically spliced into linear RNA through the removal of introns. B, CircRNAs are derived from non‐canonical splicing events. Three types of circRNA are formed. C, The biogenesis of circRNAs. CircRNAs can be miRNA sponges and modulate miRNA activity. CircRNAs can bind to RBPs and affect their functions and translocations. Certain circRNA regulates transcription and encodes proteins. In addition, circRNA‐derived pseudogenes can insert into the genome. D, The degradation of circRNA. CircRNAs are globally degraded by RNase L in early cellular innate immune responses. E, The elimination of circRNA. CircRNAs can be eliminated into the extracellular space by exosomes

CircRNAs are formed by exon back‐splicing and alternative splicing. Precursor messenger RNA (pre‐mRNA) is canonically spliced into functional linear RNA via removing introns (Figure 1). However, back‐splicing that is resistant to spliceosomes occurs in a reversed orientation to produce a single exonic or a multi‐exonic RNA molecule with a unique exon‐exon junction.^12^, ^13^ The major mechanisms of back‐splicing junction, including intron pairing‐derived circularization in which reverse complementary motifs (RCMs) specifically located in the flanking introns lead to direct base pairing to form loops, protein‐derived circularization in which a spliceosome functions via RBPs dimerization to bring the splice sites in close proximity, and lariat precursor circularization requiring at least two splicing events, are known to produce circRNAs (Figure 1).^13^, ^14^

In contrast to linear RNA, circRNA is fairly stable and resistant to exonucleases, which enables it to be accumulated in high levels, but the level of circRNA changes during the progression of disease.^15^ A study using pluripotent stem cell‐derived cardiomyocytes demonstrated that senescence, chronic stress, acute stress, oxidative stress and metabolic stress dynamically up‐regulated the expression of circRNAs like circ‐Foxo3 and circACC1.^16^, ^17^ However, the mechanism of this up‐regulation is still ambiguous. These characteristics might be associated with biogenesis and potential functions.

### 2.2 Detection of circRNAs

It is challenging to detect circRNA because their sequences are almost identical to the sequences of their linear cognate RNAs. Divergent PCR typically is used to validate circRNAs by amplifying speculative back‐spliced junction (BSJ) sites, but it can also amplify the linear RNA containing the same BSJ sequence locus. So far, the abundances and sizes of circRNAs were detected by northern blotting. Typically, Northern blot probes are labelled with the radioisotope ^32^P.^18^ Radioactive substances are potential health and environmental hazards, and they are relatively difficult to work with; therefore, non‐radioactive probes labelled with digoxigenin are suitable alternatives in the use of circRNA detection.^19^ However, although assays have been developed to detect circRNAs, further challenges include the understanding of the physicochemical nature and mechanisms of circRNA at multiple levels and the identification of circRNA binding proteins.

### 2.3 Elimination and degradation of circRNAs

To date, little is known about the elimination and degradation of circRNA in cells. Emerging studies have demonstrated that circRNAs are abundant and stable in exosomes, and can be detected in blood and urine.^20^, ^21^, ^22^ CircRNAs are eliminated into the extracellular space by exosomes that are further removed by the reticuloendothelial system or secreted by the liver and the kidney (Figure 1).^23^, ^24^ As circRNA is stable under normal conditions, the degradation of circRNA was examined by subjecting it to several stressors. It was found that the degradation of circRNA upon viral infection simulated by Poly(I:C) was triggered by the essential enzyme RNase L.^25^ Based on this finding, we predict that more degradation mechanisms of circRNA will be discovered in diverse cells and tissues.

### 2.4 Function of circRNAs

Given that circRNA is enriched in both cells and exosomes, it is possible that it could be transported outside of cells, especially because it is highly stable in both intracellular and extracellular spaces.^26^, ^27^ However, it remains a challenge to demonstrate the functional significance of circRNA because of its sequence similarity with cognate linear RNA. Presently, some functions of circRNA have been explored. CircRNAs may modulate the expression of genes and proteins via multiple pathways (Figure 1).

#### 2.4.1 circRNAs as miRNA sponges

Compared to circRNA, miRNA has been extremely well studied. miRNAs are a class of non‐coding RNAs that can bind to their target mRNAs to participate in regulation of their downstream signalling molecules.^28^ Recently, increasing evidence is suggesting that circRNAs contain binding sites of miRNA. miRNA bound by circRNA cannot bind to target mRNA and loses its capability to inhibit gene expression, resulting in an increase in mRNA. In contrast to its typical role as a miRNA sponge, circHIAT1 was shown to act as a miRNA storage device to improve the stability of mir‐195‐5p/29a‐3p/29c‐3p and partially reverse or block the migration and invasion of cancer cells.^29^ Currently, there is no compelling evidence that single circRNAs act as miRNA sponges; nevertheless, some studies have found that certain circRNAs acted as ‘collective sponges’ for a few miRNAs such as miR15/16 and let‐7. CircRNA has several binding sites for a specific miRNA to mitigate the repression of targeted mRNA.^18^ Although it has been reported that single circRNAs could be sponges for miRNA, it is considered rare because circRNAs do not typically show multiple binding sites for miRNA.^30^ Therefore, further investigation must be done to elucidate the conditions that prompt circRNAs to expose binding sites to their corresponding miRNAs.

#### 2.4.2 circRNA and RNA binding proteins

RBPs are critical for the regulation of RNA during post‐transcription, and they participate in the maturity, transport, orientation and translation of RNA. An increasing number of publications have shown that RBPs such as RNA polymerase II can bind to circRNA^4^ and impact circRNA splicing, folding, stabilization, processing and localization.^31^ CircRNAs interact with multiple proteins and subsequently influence their modes of action. RBP genes could be transcribed to numerous circRNAs that contain binding sites for the corresponding RBP. For instance, circPABPN1 blocks HuR interaction with PABPN1 mRNA by binding to HuR, leading to a low translation rate.^5^ Despite these interesting findings, the degree of detectable RBP regulation by circRNA remains an unanswered question.

#### 2.4.3 Regulating transcription

In humans, some circRNAs derived from back‐splicing (with retained introns) or from processed intron lariats remain in the nucleus.^3^, ^4^ Cellular localization seems to predict the potential function. CircRNAs located in the nucleus have been observed to be involved in regulating transcription. For instance, EIciEIF3J and EIciPAIP2, two exon‐intron circRNAs, facilitated the transcription of parental genes by interacting with U1 snRNP. Depleting EIciEIF3J and EIciPAIP2 reduced the transcription levels of the EIF3J and PAIP2 genes. This finding reveals a novel function of circRNAs localized to the nucleus: modulating gene expression.^4^

#### 2.4.4 The translation of circRNA

For many years, circRNAs were considered to be untranslatable because of no 5' 7‐methylguanosine (m7G) cap and 3' poly(A) tail. However, recent studies have indicated that a sub‐fraction of endogenous circular RNAs can be translated without the 5' 7‐methylguanosine (m7G) cap. For example, circ‐ZNF609, which is related to heavy polysomes, was translated in a cap‐independent way.^32^ In fruit fly brains, some circRNAs are translatable by showing that the UTR structures of ribo‐circRNAs (cUTRs) allow for translation without the cap.^33^ Modification of N^6^‐methyladenosine (m6A) RNA effectively triggers the translation of circRNAs into proteins in human cells. Consensus m6A motifs are enriched in circRNAs, and a single m6A site is sufficient to drive translation initiation.^34^

It is controversial whether circRNA can be directly translated. For example, circRNAs that were 126 nucleotides in length were effectively translated into proteins in *Escherichia coli* cells.^35^ Although more evidence in support of circRNA translation is needed, translation may possibly occur. In addition, it remains to be investigated whether the encoded polypeptides derived from circRNAs serve a physiological function.

#### 2.4.5 circRNA‐derived pseudogenes

It is well known that the pseudogenes derived from linear mRNAs can be retrotranscribed and integrated into host genomes. Recently, a study demonstrated that retrotranscription could also occur with circRNAs and that the processed pseudogenes were eventually inserted back into the host genome.^36^ The identification of circRNA‐derived pseudogenes suggests a novel function of circRNA: altering genomic DNA composition by inserting its retrotranscription product. Despite these findings, the function and mechanism of circRNA‐derived pseudogenes remain a mystery.

## 3 CIRCRNA AND RENAL DISEASES

CircRNA expression profiles in renal diseases such as renal cell carcinoma (RCC), diabetic nephropathy (DN), acute kidney injury (AKI) and lupus nephritis (LN) have been reported.^9^, ^11^, ^37^, ^38^ In addition, accumulating evidence has shown that circRNAs are involved in modulating inflammation, cell death and fibrosis.^39^, ^40^, ^41^ Based on these findings, circRNAs are promising therapeutic targets in renal diseases and should be intensely further investigated.

### 3.1 CircRNA and renal cell carcinoma

#### 3.1.1 Dysfunction of circRNA in renal cell carcinoma

Increasing evidence indicates that circRNAs participate in carcinogenesis, including RCC. Substantial progress has been made in the identification of differential expression of circRNAs in RCC. The interactions between circRNAs, miRNAs and genes are complex. A previous study obtained RNA microarray data from clear cell RCC (ccRCC) tissues and used control sample data from the Gene Expression Omnibus and The Cancer Genome Atlas to analyse the relationship between circRNAs, miRNAs and genes in RCC. The results revealed that 324 circRNAs were down‐regulated, whereas 218 circRNAs were up‐regulated in RCC. In addition, a circRNA‐miRNA‐mRNA interaction network was constructed.^42^

#### 3.1.2 Promising biomarkers for the diagnosis of renal cell carcinoma

CircRNAs are involved in controlling cell proliferation and have also been implicated in cancer development; they play critical roles in carcinoma progression including metastasis, invasion and drug tolerance.^6^, ^21^ RCC originating in renal tubular epithelial cells is common and causes a large number of deaths worldwide. Given the stability of circRNA, researchers are focusing on these molecules as potential biomarkers for the diagnosis and pathology of renal cancer. It was found that HHLA2 and circ‐ABCB10 were significantly up‐regulated in ccRCC tissues, and correlated with short overall survival and a poor prognosis.^43^, ^44^ Down‐regulation of Hsa‐circ‐0001451, which conspicuously increased RCC proliferation, indicated that its levels were highly correlated with RCC differentiation.^45^ CircRNA microarray analysis in ccRCC tissues and cells has indicated that significantly up‐regulated levels of circPCNXL2 are associated with poor overall survival of ccRCC patients.^8^ This evidence suggests that circRNAs may be considered robust biomarkers.

#### 3.1.3 Potential therapeutic targets of renal cell carcinoma

The functions of other circRNAs have been recently investigated. For instance, the circHIAT1/miR‐195‐5p/29a‐3p/29c‐3p/CDC42 axis has been shown to be involved in the androgen receptor (AR) promotion of RCC cell migration and invasion. Increasing circHIAT1 expression inhibited RCC progression. Knockdown of circHIAT1 decreased miR‐195‐5p/29a‐3p/29c‐3p levels, and overexpression of circHIAT1 increased miR‐195‐5p/29a‐3p/29c‐3p levels, which indicated that circHIAT1 directly interacted with miR‐195‐5p/29a‐3p/29c‐3p in RCC.^29^ Oestrogen receptor beta (ERβ) was found to suppress circATP2B1 expression, which led to reduced miR‐204‐3p and enhanced ccRCC cell invasion.^46^ In a bioinformatic analysis of RCC, a circRNA‐miRNA‐hub gene sub‐network was constructed, based on three DEcircRNAs, four DEmiRNAs and eight DEmRNAs. The possibility of an association of DEmRNA with RCC onset and progression was indicated by GO and KEGG pathway analysis.^47^ One study focused on the effects of ionizing radiation on human embryonic kidney (HEK) 293T cells and verified that 158 circRNAs were significantly differentially expressed after ionizing radiation exposure.^48^ Furthermore, cancer‐specific alternative splicing of circRNA in ccRCC has been identified; 4498 circRNA alternative splicing events were detected in the study.^49^ The function of circ‐AKT3 was confirmed to suppress ccRCC metastasis by increasing E‐cadherin expression via competitively binding miR‐296‐3p.^50^ CircPCNXL2, acting as an miRNA sponge, bound to miR‐153 to modulate ZEB2 expression in ccRCC.^8^ Furthermore, circ‐ZNF609 was significantly up‐regulated in RCC, which regulated FOXP4 expression by sponging miR‐138‐5p to promote RCC cell growth and invasion.^37^ CircC3P1 was shown to restrain RCC activity by regulating the miR‐21/PTEN axis.^51^ Based on these findings, circRNAs could have potential applications as modulators or prognostic biomarkers for RCC (Table 1).

**TABLE 1:** The potential mechanisms and target genes of circRNAs in kidney diseases

Renal disease	Functions	CircRNAs	Target miRNA	miRNA‐targeted genes
Renal cell carcinoma	As sponge	circPCNXL2^8^	miR‐153	ZEB2
	As sponge	circ‐ZNF609^37^	miR‐138‐5p	FOXP4
	miRNAs reservoir	circHIAT1^29^	miR‐195‐5p	CDC42
			miR‐29a‐3p	
			miR‐29c‐3p	
	Biomarker	HHLA2^43^		
	Biomarker	circ‐ABCB10^44^		
	Biomarker	Hsa_circ_0001451^45^		
	miRNAs reservoir	cicrATP2B1^46^	miR‐204‐3p	fibronectin 1
	As sponge	circ‐AKT3^50^	miR‐296‐3p	E‐cadherin
	As sponge	circC3P1^51^	miR‐21	PTEN
Acute kidney injury	Not clear	circ‐Dnmt3a, circ‐Akt3, circ‐Plekha7, circ‐Me1^40^		
	Biomarker	circR‐126^83^	miR‐126‐5p	
Diabetic nephropathy	As sponge	circRNA_15698^11^	miR‐185	TGF‐β1
		circLRP6^59^	miR‐205	HMGB1
Lupus nephritis	As sponge	circHLA‐C	miR‐150	SOCS1^73^
	Biomarker	hsa_circ_0000479^73^		
	Biomarker	circRNA‐002453^38^		
Hypertensive nephropathy	As sponge	circNr1h4^92^	miR‐155‐5p	Far1
Idiopathic membranous nephropathy	Biomarker	circ_101319^97^		
	Biomarker	MUC3A^98^		

### 3.2 CircRNA and diabetic nephropathy

**FIGURE 2:**
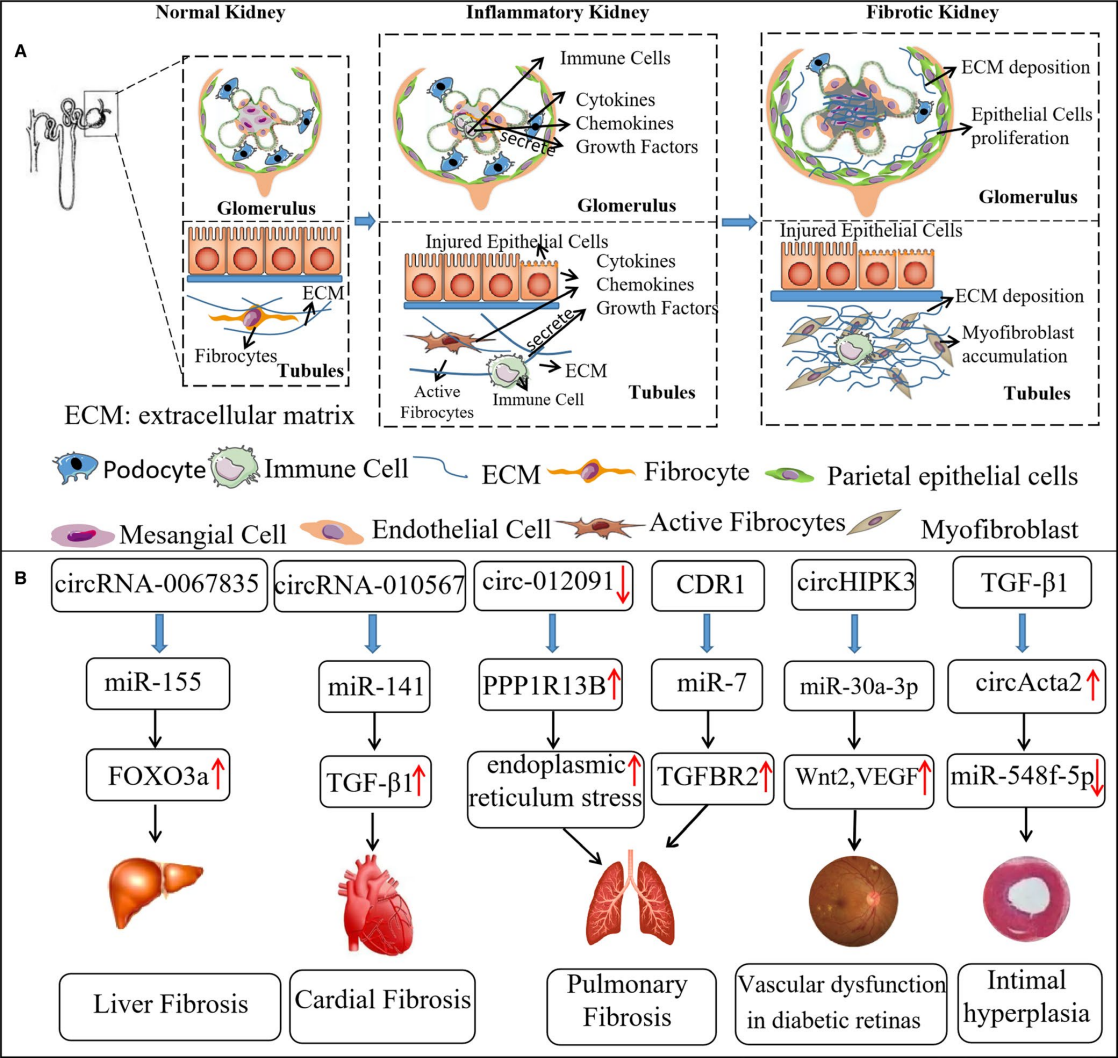
CircRNA‐mediated fibrosis and inflammation in other organs. A, Inflammation promotes progressive renal fibrosis. When renal injury occurs, circulating immune cells are recruited to the kidney and activate intrinsic kidney cells such as podocytes, which produces tissue damage factors such as cytokines, growth factors, and reactive oxygen species leading to myofibroblast accumulation and ECM production. B, CircRNA‐mediated fibrosis and inflammation in other organs or tissues. CircRNA‐mediated fibrosis has been identified in several organs such as the liver, heart, lungs, retina and vascular intima

Diabetic nephropathy (DN) is pathologically characterized by albuminuria, glomerular hypertrophy, autophagy, hypertrophy of mesangial cells and glomerular basement membrane thickening.^52^, ^53^ The prominent inflammation, observed in both the beginning and ongoing stages of kidney injury, contributes to the pathogenesis of DN.^54^, ^55^ High glucose, AGEs and oxidative stress could simultaneously induce the activation of NF‐κB to cause inflammation.^56^ Glomerular hypertrophy is associated with increases in extracellular matrix (ECM) proteins, leading to later irreversible deterioration, such as interstitial fibrosis.^57^ Recent evidence suggests that activation of Wnt/β‐catenin signalling in DN plays a pivotal role in driving renal fibrosis.^58^ Besides the non‐enzymatic glycation of proteins to form advanced glycation end products (AGEs), growth factors, such as TGF‐β1, could induce persistent inflammation and fibrosis to accelerate the progression of DN by activating downstream Smad‐dependent or Smad‐independent pathways.^7^, ^58^ Taken together, these results indicate that renal fibrosis is closely connected to renal tissue inflammation, and an excessive inflammatory response and fibrogenesis are typical features in DN (Figure 2A).

Emerging evidence indicates the role of circRNAs in DN. For instance, a recent study confirmed that circRNA‐15698 is highly expressed in both DN mice and mouse mesangial cells. When exposed to high glucose levels, the levels of fibrosis‐related proteins, such as collagen type I (Col. I), and Col. IV were significantly increased. The knockdown of circRNA‐15698 by small interfering RNAs (siRNAs) led to a significant decrease in these fibrosis‐related proteins. Mechanistically, circRNA‐15698 acted as an miR‐185 sponge, increasing TGF‐β1 protein levels and stimulating ECM‐related protein synthesis in DN.^11^ Furthermore, one recent study suggested that up‐regulated circLRP6 modulated the mesangial cell injury by sponging miR‐205 under high glucose conditions. In this study, Chen et al reported that circLRP6 regulated high glucose‐induced proliferation, oxidative stress, ECM accumulation, inflammation in mesangial cells and activation of the TLR4/NF‐κB pathway.^59^ Taken together, fibrotic and inflammatory mechanisms play an important role in DN development and progression. Although there are few studies on circRNA in DN, the present data propose that circRNAs, acting as miRNA sponges, might be involved in pathophysiological processes in DN (Table 1).

Although the roles of circRNA in inflammation and fibrosis in DN remain to be discovered, their roles in regulating inflammation and fibrosis in other organs or tissues, including the liver, lungs, heart and blood vessels, have been demonstrated (Figure 2B). Considering their targets, such as TGF‐β1, also function in DN, these studies may act as references for the future study of DN.^60^, ^61^, ^62^, ^63^, ^64^ For example, circHIPK3 expression was significantly up‐regulated in diabetic retinas and, acting as an endogenous miR‐30a‐3p sponge, led to increased vascular endothelial growth factor‐C and Wnt2 expression, which contributed to inflammation and vascular dysfunction.^65^ TGF‐β–mediated induction of circActa2 promotes α‐SMA protein expression via sponging of miR‐548f‐5p in vascular smooth muscle cells.^66^ CircRNA CDR1 was found to act as an miR‐7 sponge to up‐regulate TGFBR2, which primarily functions during pulmonary fibrosis progression by stimulating the process of epithelial‐mesenchymal transition (EMT).^41^ CircRNA_010567 acted as a sponge of miR‐141, which binds to TGF‐β1 to regulate fibrosis‐associated proteins in cardiac fibroblasts.^7^ All these findings suggest that circRNAs might be key regulators in the pathogenesis of DN.

Of note, many important questions about circRNAs in DN remain to be answered. For example, as the current evidence was mainly derived from mesangial cells, what are the functions of circRNAs in podocytes? Additionally, up until now, the functions of circRNAs have not been validated in vivo. These points highlight the requirement for further analyses.

### 3.3 CircRNA and lupus nephritis

Systemic lupus erythematosus (SLE) is a chronic autoimmune disease accompanied by the production of numerous abnormal antibodies, leading to various manifestations of the disease. In recent years, increasing studies have focused on the expression and roles of circRNAs in SLE. For example, decreased circRNA in the peripheral blood mononuclear cells of SLE patients was investigated.^25^ CircRNA microarray analysis showed that 113 up‐regulated and 94 down‐regulated circRNAs were detected in plasma samples derived from SLE patients.^67^ Emerging evidence indicates that circRNAs are involved in the pathophysiology of SLE.^68^, ^69^ Collectively, these data suggest that circRNAs could be used as potential biomarkers or therapeutic targets for SLE.

Although SLE is an autoimmune disease that could damage any organ, it commonly attacks the kidney. LN is a primary cause of morbidity and mortality in SLE.^70^ Typically, LN occurs in individuals with a specific genetic background, facilitated by certain environment conditions. To date, multiple circRNAs have been associated with LN susceptibility. For instance, 116 differentially expressed circRNAs, including 41 up‐ and 75 down‐regulated circRNAs, have been demonstrated in severe LN when compared with mild LN, while miR‐210‐5p has been verified as a target of mmu_circRNA_34428 in LN.^71^ A recent study verified 171 diversely expressed circRNAs in LN renal biopsies. CircHLA‐C was highly up‐regulated, but miR‐150 was down‐regulated in patients with LN, compared with healthy controls. A positive correlation between miR‐150 and renal chronicity index was found in LN patients, while circHLA‐C seems to be negatively correlated with miR‐150.^10^ In another study, miR‐150 attenuated expression of the antifibrotic protein suppressor of cytokine signalling 1 (SOCS1) and promoted renal fibrosis in LN.^72^ In addition, the circRNA‐002453 level was highly elevated in plasma derived from LN patients compared to SLE patients without LN, rheumatoid arthritis patients and healthy controls. In particular, its expression showed a positive correlation with 24‐hour proteinuria and renal SLE disease activity index scores (Table 1).^38^ Recently, a bioinformatic analysis showed that high hsa_circ_0000479 levels were correlated with low albumin levels, positive urine protein and low haemoglobin, which indicated that it might be involved in SLE‐associated renal injury.^73^ Although evidence of the molecular mechanisms of circRNAs in LN is currently lacking, future investigations will identify and illuminate the functions of circRNAs in LN and eventually contribute to its diagnosis and treatment.

### 3.4 CircRNA and acute kidney injury

AKI, characterized as a rapid decline in renal function, is a severe complication in critically ill patients and has been identified as an independent risk factor concerning survival.^74^, ^75^ Accumulating evidence shows that the major pathological features of AKI include oxidative stress, inflammation and programmed cell death of renal tubular epithelial cells.^76^, ^77^ Many key pathways or modulators, such as the NF‐κB–mediated pro‐inflammatory pathway, the caspase‐correlated apoptosis pathway and TGF‐β/Smad signalling, are involved in the pathophysiological process of AKI.^78^, ^79^ Unfortunately, specific therapy for AKI is still lacking.^74^ Recently, circRNAs have become the focus of research, as they might have potential in the diagnosis, as well as the treatment, of AKI.

CircRNAs were dysregulated in types of AKI models induced by nephrotoxic agents and LPS. Differentially expressed circRNAs have been associated with various biological processes, including cellular component organization and biogenesis, localization, and other processes during AKI. RNA‐Seq results demonstrated that 1664 circRNAs were significantly highly expressed and 474 circRNAs were uniquely expressed in the kidney.^80^ Thirty‐four up‐regulated and 22 down‐regulated circRNAs were verified in mouse kidneys of IR‐induced AKI models.^9^ Three hundred and sixty‐eight circRNAs were found to be differentially expressed in response to cisplatin‐induced AKI.^81^ Additionally, the study revealed that the expression of 38 circRNAs was significantly dysregulated in contrast‐induced AKI.^82^ The expression of circ‐Dnmt3a, circ‐Akt3, circ‐Plekha7 and circ‐Me1 was in the top 20 most significantly differentially expressed circRNAs, and was predicted to be associated with PI3K‐Akt signalling in AKI.^40^ Serum circR‐126, which may act as a biomarker for predicting the mortality of AKI via miR‐126‐5p sponging, was elevated in AKI patients.^83^ CircANRIL promoted inflammation and apoptosis of HK2 cells treated with LPS, via miR‐9/NF‐κB pathways.^84^ Moreover, the functions of circRNAs in modulating programmed cell death and cell cycle progression indicate that they could be novel potential regulators of AKI.^21^ For instance, mmu‐circRNA015947 interacted with miRNAs to induce downstream gene expression and, as a result, participated in apoptosis‐correlated pathways, which are involved in the pathogenesis of IR injury.^85^ Furthermore, by modulating miR‐671, circRar1 induced the transcriptional activity of apoptosis‐linked factors, such as caspase‐8, in lead‐induced neurotoxicity.^86^

CircRNAs demonstrate great promise as disease biomarkers due to high resistance to exonucleases, and they might even be highly accumulated. Their putative function as miRNA sponges makes them particularly interesting therapeutic targets for future research. Despite these findings, the understanding of the function of circRNA in AKI is at a rather early stage, and further investigation is required to explore the modulation of circRNAs during AKI progression, including during injury, repair and AKI‐to‐chronic kidney disease (CKD) transition, and to verify circRNAs that are specifically expressed in the kidney. Such circRNAs could be used as biomarkers for the diagnosis of AKI (Table 1). Whether they might serve as therapeutic targets will no doubt be the patient of future research.

### 3.5 CircRNA and kidney calculi

Kidney calculi are common chronic renal disease, and approximately 1 in 17 Chinese adults suffer from this illness.^87^ Patients with kidney calculi generally present with pain and urinary tract infections that can result in the gradual loss of kidney function.^88^ A recent study found that 145 circRNAs, including 58 up‐regulated circRNAs, were differentially expressed in urolithiatic rat kidneys.^89^ Rno‐miR‐138‐5p and rno‐miR‐672‐5p, which are co‐expressed with miRNAs, have been proven to be involved in kidney calculi.^89^ Calcium phosphate crystals are abnormally deposited in the vessel walls, leading to vascular calcification, a common complication of CKD. This study showed that circSamd4a exerts an anti‐calcification role, via functioning as an miRNA sponge in a vascular calcification model, to reduce the complications of CKD.^90^ To date, although information regarding the roles of circRNAs in kidney calculi is scarce, these findings present a novel perspective on the potential action of circRNA in kidney calculi progression.

### 3.6 CircRNA and hypertensive nephropathy

Hypertension is a common chronic disease that presents with high arterial pressure and may be accompanied by pathological changes in vital organs such as the heart, kidney and blood vessels. Evidence shows that certain circRNAs play a role in the pathological development of hypertension and hypertensive nephropathy. A total of 12 846 circRNAs have been identified in the rat kidney. A total of 318 circRNAs are differentially expressed in the Dahl salt‐sensitive rat versus the Dahl salt‐resistant rat, and 110 circRNAs are differentially expressed in the spontaneously hypertensive rat versus the Wistar Kyoto rat.^91^ Researchers analysed circRNA differential expression and also found that multiple circRNAs were altered in the kidneys of rats with hypertensive nephropathy; 124 differentially expressed rat renal circRNAs were discovered in hypertensive rats with kidney injury.^92^ A recent study demonstrated that hsa_circ_0014243 was elevated in blood samples derived from hypertensive patients, and it could be used as a diagnostic marker for hypertension.^93^ CircNr1h4 derived from the Nr1h4 gene was confirmed to modulate renal injury in hypertensive mice by targeting miR‐155‐5p.^92^ Levels of circulating miR‐103a‐3p were elevated in both hypertensive nephropathy patients and angiotensin II‐infused mice, and resulted in renal inflammation and fibrosis by acting on NF‐κB/p65.^94^ So far, no reference to circRNA sponging of miR‐103a‐3p in kidney diseases has been reported, but the down‐regulation of miR‐103a‐3p by circTCF25, and the promotion of proliferation and migration have been reported in bladder cancer.^95^ Circulating miR‐103a‐3p levels are tightly linked to hypertensive nephropathy^94^; therefore, focusing on its corresponding circRNA might be a novel therapy target for hypertensive nephropathy.

### 3.7 CircRNA and idiopathic membranous nephropathy

Idiopathic membranous nephropathy (IMN), regarded as an organ‐specific autoimmune disease, is one of the major causes of nephritic syndrome in adults and is an important factor in the recurrence of nephritic syndrome in patients after renal transplantation.^96^ As the pathogenesis of the disease is not fully understood, and because few sensitive biomarkers have been found that reflect disease activity, an effective treatment for IMN is lacking in modern medicine.^97^ It has recently been found that circRNAs may play a function in the occurrence and development of IMN. For instance, one study examined expression levels of circRNAs in the peripheral blood of patients with IMN. The results showed that a total of 955 differentially expressed circRNAs were found in blood samples, 645 of which were up‐regulated and 310 of which were down‐regulated. The levels of circ_101319 were significantly higher and exhibited promising diagnostic value in patients with IMN.^97^ Another study demonstrated differential expression of circular RNAs in exosomes from serum and urine in patients with IMN. This verified that MUC3A might be considered as a potential biomarker for the diagnosis of IMN.^98^ Taken together, these data provide an insight into the pathogenesis of IMN and a possible solution for future diagnosis and treatment.

## 4 CONCLUSION

The exploration of abundant circRNAs has expanded our understanding of the diversity of gene expression. Differently expressed circRNAs have been observed in a variety of normal tissues and diseases, which indicates that circRNAs may have vital physiological and pathological function.^99^ CircRNAs play key roles in gene expression regulation and biological processes, mainly through the regulation of miRNA and target gene expression.^2^ In addition, circRNA‐derived pseudogenes have been found to integrate into genomic DNA and alter its composition.^36^ However, knowledge about the functions and mechanisms of circRNA‐derived pseudogenes is limited, and more work should be done in the future. In addition, circRNA have been identified as novel therapeutic targets and biomarkers for renal disease. Future studies should confirm the exact mechanisms through which circRNAs interact with specific proteins and other non‐coding RNAs to mediate their effects. Recently, the mechanism of circRNA degradation was revealed by treating it with a number of stressors.^25^

Despite recent discoveries in circRNA biogenesis and function, many challenges remain to be overcome. For example, few technologies exist for the investigation of the physicochemical properties and mechanisms of circRNAs. Moreover, we lack an understanding of how to further analyse kidney‐derived samples to verify circRNAs related to renal disease. Though many circRNAs have been confirmed in kidney tissue, their expression profiles and potential roles during disease development and its inhibition or stimulation remain largely unknown. Increasing exploration into the predictive roles and functions of circRNAs in renal diseases, especially testing the therapeutic potential of circRNAs in animal models, will contribute to the better understanding of the pathophysiological and physiological processes of the kidney, and this will hopefully become an intense area of research.

## AUTHORS CONTRIBUTION

Xiao‐ming Meng provided direction and guidance throughout the preparation of this manuscript. Juan Jin, Haolu Sun and Chao Shi wrote and edited the manuscript. Hui Yang, Yiwan Wu and Wanhai Li reviewed and made significant revisions to the manuscript. Yu‐hang Dong and Liang Cai collected and prepared the related papers. All authors read and approved the final manuscript.

## CONFLICT OF INTERESTS

The authors declare that they have no conflict of interests.

## Data Availability

The data used to support the findings of this study are included within the article.
